# Identification of Key Candidate Genes and Pathways in Colorectal Cancer by Integrated Bioinformatical Analysis

**DOI:** 10.3390/ijms18040722

**Published:** 2017-03-28

**Authors:** Yongchen Guo, Yonghua Bao, Ming Ma, Wancai Yang

**Affiliations:** 1 Institute of Precision Medicine, Jining Medical University, Jining 272067, China; guoyongchen2005@126.com (Y.G.); baoyonghua2005@126.com (Y.B.); 2 Department of Surgery, Jining Medical University, Jining 272067, China; mingma17@163.com; 3 Department of Pathology, University of Illinois at Chicago, Chicago, IL 60612, USA

**Keywords:** colorectal cancer, bioinformatical analysis, differentially expressed genes

## Abstract

Colorectal cancer (CRC) is one of the most common malignant diseases worldwide, but the involved signaling pathways and driven-genes are largely unclear. This study integrated four cohorts profile datasets to elucidate the potential key candidate genes and pathways in CRC. Expression profiles GSE28000, GSE21815, GSE44076 and GSE75970, including 319 CRC and 103 normal mucosa, were integrated and deeply analyzed. Differentially expressed genes (DEGs) were sorted and candidate genes and pathways enrichment were analyzed. DEGs-associated protein–protein interaction network (PPI) was performed. Firstly, 292 shared DEGs (165 up-regulated and 127 down-regulated) were identified from the four GSE datasets. Secondly, the DEGs were clustered based on functions and signaling pathways with significant enrichment analysis. Thirdly, 180 nodes/DEGs were identified from DEGs PPI network complex. Lastly, the most significant 2 modules were filtered from PPI, 31 central node genes were identified and most of the corresponding genes are involved in cell cycle process, chemokines and G protein-coupled receptor signaling pathways. Taken above, using integrated bioinformatical analysis, we have identified DEGs candidate genes and pathways in CRC, which could improve our understanding of the cause and underlying molecular events, and these candidate genes and pathways could be therapeutic targets for CRC.

## 1. Introduction

Colorectal cancer (CRC) is one of the most common malignancies in the world, there were estimated more than 777,000 of new cases in 2015 in the developed countries [1,2], and in China alone, there were about 376,000 of new CRC cases and 191,000 of death CRC cases in 2015, accounting for the fifth of malignant tumor incidence and mortality [3]. Although there are extensive studies on the mechanism in colorectal cancer formation and progression, the causes of colorectal cancer is still not clear. The occurrence and progression of colon cancer are correlated with multiple factors from the point of view of science and research, for instance, gene aberrations, cellular and environmental factors [4]. Due to high morbidity and mortality in colorectal cancer, revealing the causes and the underlying molecular mechanisms, discovering molecular biomarkers for early diagnosis, prevention and personalized therapy, is critically important and highly demanded.

Gene chip or gene profile is a gene detection technique that has been used for more than ten years, using gene chips can quickly detect all the genes within the same sample time-point expression information, which is particularly suitable for differentially expressed genes screening [5]. With the wide application of gene chips, a large number of cores slice data have been produced, and most of the data have been deposited and stored in public databases. Integrating and re-analyzing these data can provide valuable clues for new research. Many microarray data profiling studies have been carried out on CRC in recently years [6], and hundreds of differentially expressed genes (DEGs) have been obtained. However, the results are always limited or inconsistent due to tissue or sample heterogeneity in independent studies, or the results are generated from a single cohort study. Thus, no reliable biomarkers have been identified in CRC. However, the integrated bioinformatics methods combining with expression profiling techniques will be innovative and might solve the disadvantages.

In this work, we have downloaded four original microarray datasets GSE28000 [7], GSE21815 [8,9], GSE44076 [10,11], GSE75970 (unpublished data), from NCBI-Gene Expression Omnibus database (NCBI-GEO) (Available online: https://www.ncbi.nlm.nih.gov/geo), from which there are total of 319 CRC cases and 103 normal colon mucosa data available. We filtered DEGs in Morpheus Website with data processing standard, and subsequently, developed Gene ontology and pathway enrichment analysis for screening of DEGs with DAVID, KEGG PATHWAY (Available online: http://www.genome.jp/kegg), Reactome (Available online: http://www.reactome.org), BioCyc (Available online: http://biocyc.org), Panther (Available online: http://www.pantherdb.org) [12], NHGRI and Gene Ontology online website, developed integration of DEGs protein–protein interaction (PPI) network (Available online: http://string-db.org) and modular analysis to identify hub genes in CRC. Identifying DEGs and enriching their biological functions and key pathways will provide more accurate, practically reliable biomarkers for early diagnosis and individualized prevention and therapy.

## 2. Results

### 2.1. Identification of DEGs in Colorectal Cancers

**Figure 1:**
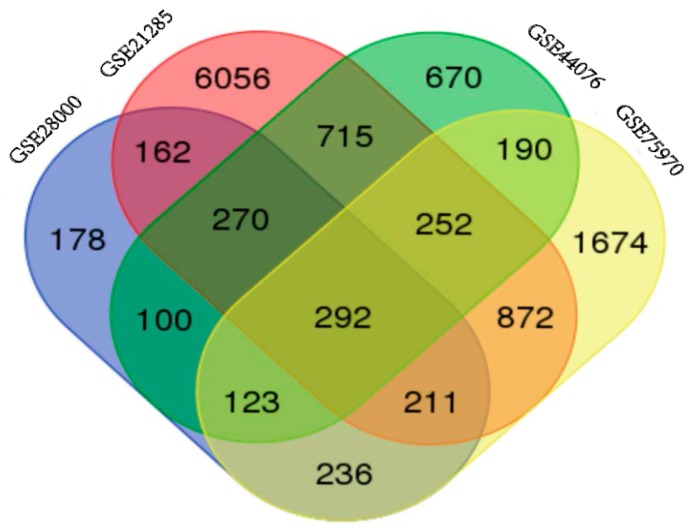
Identification of 292 commonly changes DEGs from the four cohort profile data sets (GSE28000, GSE21815, GSE44076 and GSE75970) using Morpheus Website (Available online: https://software.broadinstitute.org).Different color areas represented different datasets. The cross areas meant the commonly changed DEGs. DEGs were identified with classical *t*-test, statistically significant DEGs were defined with *p* < 0.05 and [logFC] > 1 as the cut-off criterion.

NCBI-GEO is a free database of microarray/gene profile and next-generation sequencing, from which colorectal cancer and normal or adjacent mucosa tissue gene expression profile of GSE28000, GSE21815, GSE44076 and GSE75970 were obtained. The microarray data of GSE28000 had 43 American Africans (AA) and 43 European Africans (EA) CRC tissues and 40 normal colon tissues [7]. The GSE21815 data had 131 CRC tissue and 9 normal colon tissue [8,9]. The GSE44076 data included 98 CRC tissues and 50 healthy colon mucosa [10,11], and the GSE75970 data had 4 pairs of colorectal cancer tissues and matched paraneoplastic tissues. Using *p* < 0.05 and [logFC] > 1 as cut-off criterion, we extracted 1572, 8830, 2612 and 3850 differentially expressed genes (DEGs) from the expression profile datasets GSE28000, GSE21815, GSE44076 and GSE75970, respectively. After integrated bioinformatical analysis, total of 292 consistently expressed genes were identified from the four profile datasets (Figure 1), including 165 up-regulated genes and 127 down-regulated genes in the colorectal cancer tissues, compared to normal colon tissues (Table 1). Employing Morpheus software, we developed a heat map of the 165 up-regulated and 127 down-regulated DEGs using data profile GSE44076 as a reference, showing the significantly differential distribution of the 292 DEGs (Figure S1).

### 2.2. DEGs Gene Ontology Analysis in Colorectal Cancers

**Figure 2:**
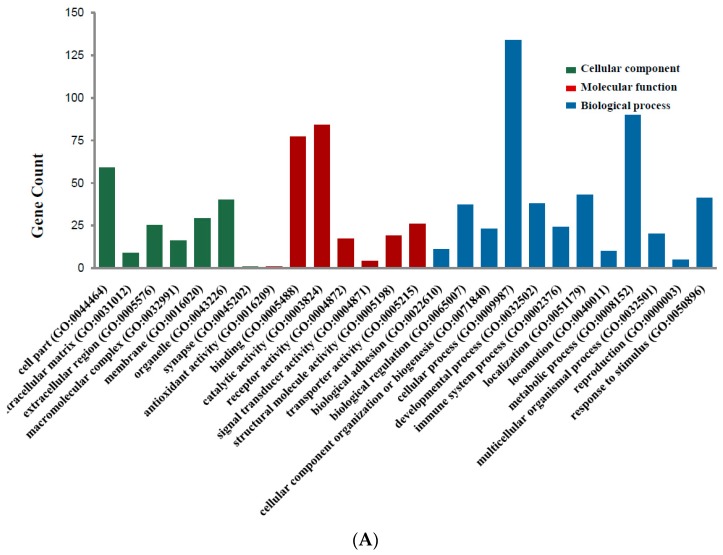

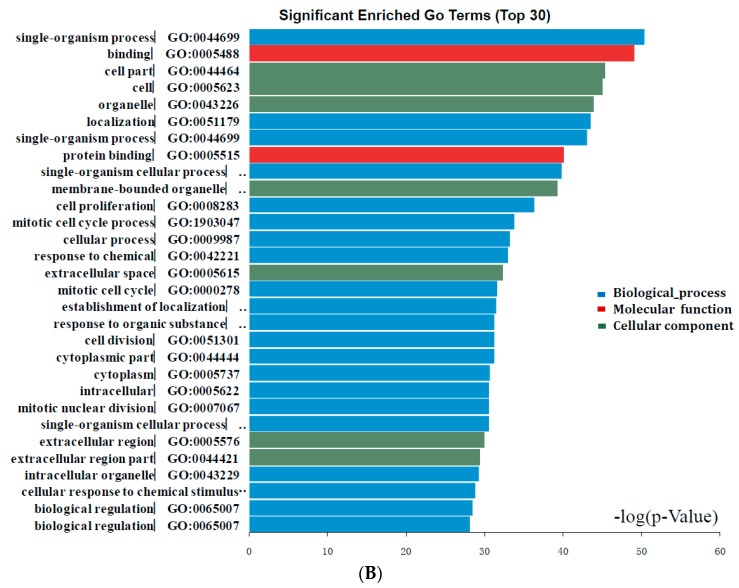
Gene Ontology analysis and significant enriched GO terms of DEGs in colorectal cancer. (**A**) GO analysis classified the DEGs into 3 groups (i.e., molecular function, biological process and cellular component); (**B**) Significant Enriched Go Terms of DEGs in colorectal cancer based on their functions.

Candidate DEGs functions and pathways enrichment were analyzed using multiple online databases, including DAVID, KEGG PATHWAY (Available online: http://www.genome.jp/kegg), Reactome (Available online: http://www.reactome.org), BioCyc (Available online: http://biocyc.org), Panther (Available online: http://www.pantherdb.org) [12], NHGRI and Gene Ontology website with *p* < 0.05 as the cut-off criterion [13,14,15]. DEGs gene ontology analysis (GO) were performed with DAVID, Panther. The DEGs were classified into three functional groups: molecular function group, biological process group, and cellular component group (Figure 2A). As shown in Figure 2B and Table 2, in the biological process group, up-regulated genes mainly enriched in single-organism process, single-organism cellular process, cell proliferation process and mitotic cell cycle process, and the down-regulated genes mainly enriched in localization, single-organism process, single-organism cellular process, mitotic cell cycle process. In the molecular function group, up-regulated genes mainly enriched in binding, receptor binding, protein binding, and the down-regulated genes mainly enriched in binding, protein binding. In the cellular component group, up-regulated genes mainly enriched in extracellular space, extracellular region, and the down-regulated genes mainly enriched in cell part, membrane-bounded organelle, organelle. These results showed that most of the DEGs were significantly enriched in single-organism, binding, cell part and mitotic cell cycle.

### 2.3. Signaling Pathway Enrichment Analysis

**Figure 3:**
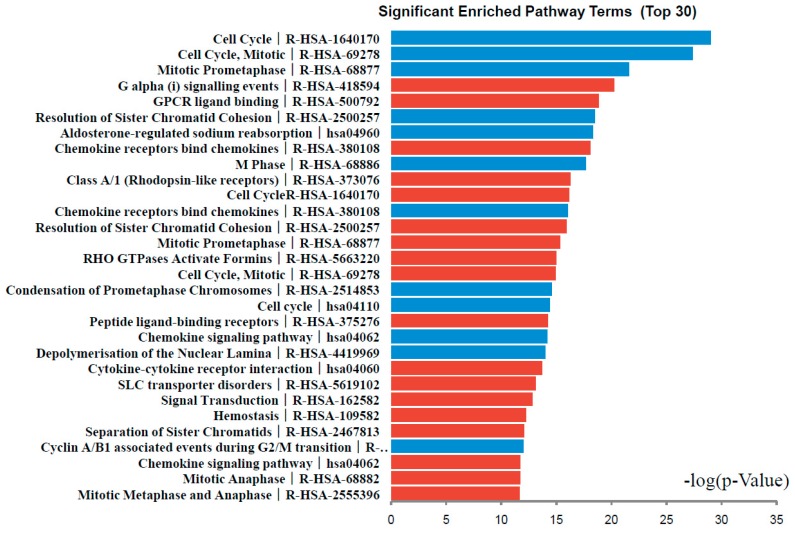
Significantly enriched pathway terms of DEGs in colorectal cancer. DEGs functional and signaling pathway enrichment were conducted using online websites of KEGG PATHWAY, Reactomen, BioCyc, Panther, NHGRI and Gene Ontology analysis.

DEGs functional and signaling pathway enrichment were conducted using online websites of KEGG PATHWAY, Reactomen, BioCyc, Panther, NHGRI and Gene Ontology. The up-regulated genes mainly enriched in G α (i) signaling events, GPCR ligand binding, Chemokine receptors bind chemokines, Class A/1 (Rhodopsin-like receptors), and Cell Cycle signaling pathway. The down-regulated genes mainly enriched in Cell Cycle, Mitotic Prometaphase, Resolution of Sister Chromatid Cohesion, Aldosterone-regulated sodium reabsorption, and Chemokine receptors bind chemokines signaling pathway (Figure 3, Table 3). Signaling pathway analysis showed that DEGs had common pathways in Cell Cycle, Chemokine receptors bind chemokines.

### 2.4. Key Candidate Genes and Pathways Identification with DEGs Protein–Protein Interaction Network (PPI) and Modular Analysis

**Figure 4:**
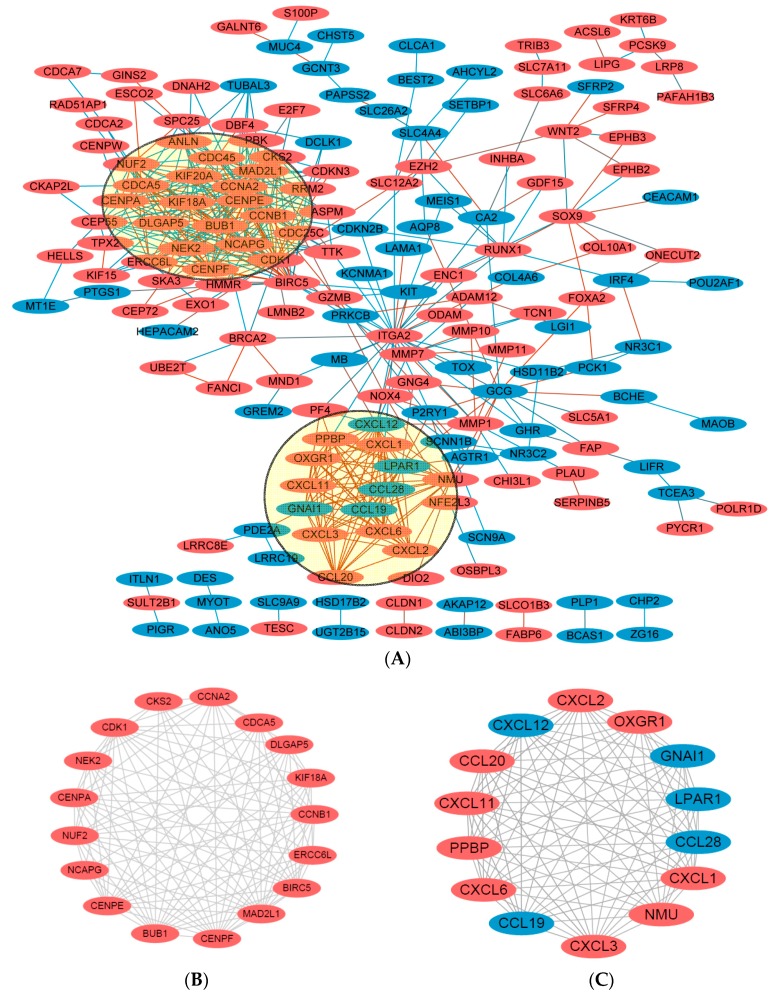
DEGs protein–protein interaction (PIP) network complex and modular analysis. (**A**) Using the STRING online database, total of 180 DEGs (113 up-regulated in Orange standing for upregulationand 67 down-regulated genes in Blue standing for downregulation) were filtered into the DEGs PPI network complex. The two highlighted circle areas were most significant modules; (**B**) Module 1 consists of 17 nodes and 109 edges, which are mainly associated with cell cycle process, sister chromatid and segregation; (**C**) Module 2 consists of 14 nodes and 91 edges, which are mainly associated with chemokine signaling pathway, Gα (i) signaling events, and GPCR ligand binding pathway.

Using the STRING online database (Available online: http://string-db.org) [16] and Cytoscape software [17], total of 180 DEGs (113 up-regulated and 67 down-regulated genes) of the 292 commonly altered DEGs were filtered into the DEGs PPI network complex, containing 180 nodes and 518 edges (Figure 4A), and 112 of the 292DEGs did not fall into the DEGs PPI network. Among the 180 nodes, 31 central node genes (bold in Table 1) were identified with the filtering of degree >10 criteria (i.e., each node had more than 10 connections/interactions) , and the most significant 10 node degree genes were *CDK1*, *CCNB1*, *CENPE*, *KIF20A*, *CXCL12*, *DLGAP5*, *CCNA2*, *ITGA2*, *MAD2L1* and *NMU.* According the degree of importance, we chose 2 significant modules from the PPI network complex for further analysis using Cytotype MCODE. Pathway enrichment analysis showed that Module 1 consisted of 17 nodes and 109 edges (Figure 4B, Table 4), which are mainly associated with cell cycle process, sister chromatid and segregation, and that Module 2 consisted of 14 nodes and 91 edges (Figure 4C, Table 5), which are mainly associated with chemokine signaling pathway, Gα (i) signaling events, and GPCR ligand binding pathway. 

### 2.5. Validation of the DEGs in TCGA Dataset

To confirm the reliability of the identified DEGs from the 4 datasets, we downloaded the TCGA CRC dataset (including 22 normal and 122 colon cancers) and analyzed the data using the same strategy as used in this report. We found that all the 165 up-regulated genes identified in this study were also significantly overexpressed in the TCGA colon cancers (See Figure S2A and Table S1), and 114 down-regulated genes identified in this study were also found significantly under-expressed in the TCGA colon cancers (See Figure S2B and Table S1), only 13 of the downregulated genes were not in the list. The total consistency of the up- and down-regulated genes was 95.5%, suggesting our results of the identified candidate genes are reliable.

## 3. Discussion

Numerous basic and clinical studies have been conducted to reveal the causes and underlying mechanisms of colorectal cancer formation and progression in the past several decades, but the incidence and mortality of CRC is still very high in the world, because most studies focus on a single genetic event or the results are generated from a single cohort study [18]. This study integrated four cohorts profile datasets from different groups, utilized bioinformatics methods to deeply analyze these datasets, and identified 292 commonly changed DEGs(165 up-regulated and 127 down-regulated) in the first step. In the second step, the 292 EDGs were classified into three groups (molecular functions, biological process and cellular component groups) by GO terms using multiple approaches.In the third step, the up- and down-regulated DEGs were further clustered based on functions and signaling pathways with significant enrichment analysis.In the fourth step, DEGs protein–protein interaction (PPI) network complex was developed and 180 nodes/DEGs were identified with 518 edges, and finally, the most significant 2 modules were filtered from the PPI network complex, among them, 31 central node genes were identified and most of the corresponding genes were associated with cell cycle process, Chemokines and G protein-coupled receptor signaling pathways.

It has been known that Wnt/β-catenin activation and malignant transformation of inflammatory bowel disease are the two major causes of colorectal cancer. Both Wnt/β-catenin [19,20] and inflammatory signaling pathway activation [21] lead to intestinal epithelial disruption ofhomeostasis, for instance, proliferation is increased, differentiation and apoptosis are decreased, in the intestinal tract.Through integrated bioinformatical analysis, we have identified 31 central node/genes, among them, the first module or cluster (Figure 4B) consisting of 17 genes, including *CDK1*, *CCNB1*, *CENPE*, *KIF20A*, *CCNA2*, *MAD2L1*, were listed at the top of the most changed genes, and their biological functions are involved in cell cycle control and mitotic regulation [22]. *CCNB1* and *CCNA2* are cyclins B and A, and *CDK1* is cyclin-dependent kinase 1, all three of them are indirect downstream targets of β-catenin and exhibit opposite functions with p21CIP1 and p27KIP1 to promote cycle and mitosis [23,24]. In contrast, p21CIP1 and p27KIP1 are cyclin-dependent kinase inhibitors and delay cell cycle and mitosis, and both p21CIP1 and p27KIP1 bind to cyclin D1, a direct downstream target of β-catenin [23,24]. Besides the functions of promoting cell cycle, mitosis and cell proliferation to initiate colorectal cancer development, Wnt/β-catenin signaling pathway also involves in epithelial-mesenchymaltransition (EMT), the later is an early event of invasion and metastasis, through up-regulating EMT-associated transcription factors Snail, Twist and ZEB1 [4,25]. In addition, Wnt/β-catenin signaling keeps the stemness of cancer stem cells via up-regulating stem cell-related transcription factors *Oct4*, *Sox2*, *c-Myc*, *Nanog* and *KLF4* [4,25]. Cancer stem cells are the main causes of cancer cell proliferation, metastasis and therapeutic resistance. 

The second module/cluster (Figure 4C) is mainly consisted of C-X-C Motif Chemokine Ligend (CXCL) family members *CXCL1*, *CXCL2*, *CXCL6*, *CXCL8* and *CXCL12*. The up-regulation of chemokine and cytokines expression are the major characteristics of inflammation-associated colorectal cancer [26,27]. The studies from us and others have demonstrated that the increase of cytokines (e.g., COX2, NF-κB, TNFα, IL-1β, IL6, etc.), CXCLs and CXC receptors (CXCRs) could be the cause of malignant transformation of chronic colitis, which shows defect of mucosa barrier, causing gut microbiota disorder and enhancement of inflammation, resulting in genetic mutations, epigenetic alteration (e.g., DNA methylation, histone acetylation and miRNAs, etc.) and oncogenic signaling activation (e.g., Wnt, Ras, PI3K/AKT/STAT3, etc.) [21,28,29,30]. The changes of genetic and epigenetic alterations and oncogenic signaling activation lead to colorectal carcinogenesis and progression. Moreover, the studies have also shown that CXCL/CXCR and PI3K/AKT/STAT3 signaling pathways participate in the regulation of EMT- and cancer stem cell- associated events, and up-regulate the expression of Snail, Twist, ZEB1, Oct4, Sox2, c-Myc, Nanog and KLF4 in colorectal cancers [25]. 

Besides the chemokine signaling in module/cluster 2 (Figure 4C), Gα (i) signaling and G-protein coupled receptor (GPCR) signaling pathway is also in this module. Previous studies have demonstrated that GPCR is associated with metabolism, and the abnormal metabolism leads to chronic inflammation in the gut and oncogenic signaling activation [21,31]. In addition, GPCR participates in Rho-GTPase pathway and modulates cell-cell contact proteins, affects cell morphology and mobility [32,33]. Recent studies have also shown that aberrant expression of GPCR up-regulate the expression of intestine-specific stem cell marker LGR5 [34], causing colorectal carcinogenesis in vivo and chemo-resistance in vitro. 

Consistent with our studies, the recent studies have also reported the identification of DEGs in colorectal cancers [35,36]. For examples, Yan’s groups analyzed gene expression profile GSE32323 that contained 34 samples, including 17 specimens of CRC tissues and 17 of paired normal tissues from CRC patient. From which, 1347 DEGs were identified, including 659 upregulated genes and 688 downregulated genes, the majority of the identified DEGs were mainly enriched in cell cycle-related biological processes and pathways, such as *CDK1*, *CCNB1*, *MAD2L1*, *BUB1B*, etc. These DEGs were as also identified in our study. We wanted to point out that Yan’s study was based on a single dataset generated from Japanese patients, and the main reason for Japanese colorectal carcinogenesis is the alteration of cell cycle-related biological processes and pathways [37]. Different from Yan's report, we used 4 datasets generated from the African American, European American, Spanish, Japanese and Chinese, represented 5 different races of world-wide populations, and we found that besides the cell cycle-related biological processes and pathways, inflammatory pathway (CXCL-CXCR), another major reason for colorectal carcinogenesis, was identified, because one of the major causes of CRC in European Americans and Spanish is malignant transformation of chronic colitis [21]. 

Another report from Kou et al. was based on the single dataset GSE4107 generated from 12 CRC patients and ten healthy controls [36,38]. A total of 612 up- and 639 downregulated genes were identified. The upregulated DEGs were mainly involved in the regulation of cell growth, migration, and the MAPK signaling pathway. The downregulated DEGs were significantly associated with oxidative phosphorylation, Alzheimer's disease, and Parkinson’s disease. It should be noticed that these CRC patients were from early onset CRC withnon-FAP (familial adenomatous polyposisnon) or non-hereditary non-polyposis and all patients were young (age < or = 50 years old), ethnicity- (Chinese), and tissue-matched [38]. One of the pathways, that is G protein-coupled receptor pathway, was also identified in our study, even though our conclusion was generated from 4gene expression profile datasets from middle- or late-stage with very few early stages of CRC. 

Colorectal cancer is a group of histologically and molecularly heterogeneous diseases characterized by differing sets of epigenetic and genetic alterations involved in multiple functional signaling pathways, and the later is modulated by genetic and epigenetic events, leading to the alterations of gene expression at transcriptional and/or translational levels. Therefore, the characteristics of CRC cannot be explained only by gene expression profiles, but gene expression profile could represents one type of alterations in cancer or characteristics of cancers. Many factors should be considered, including gene mutations, hypo- or hyper-methylation, microRNAs and long non-coding RNAs, MSI, etc., which could cause or participate in colorectal carcinogenesis and are associated with survival in part or together. For instance, the association of LINE-1 hypomethylation with inferior survival is stronger in MSI-high CRCs than in MSS CRCs [39]. Moreover, the molecular classification of cancer types or subtypes have been identified according to the molecular changes (e.g., gene expression, epigenetic changes, etc.). In the case of colorectal cancer, the CRC could be classified into adenocarcinoma, mucinous adenocarinoma, signet-ring cell carcinoma, according to cell components of the CRC tissues, and the components of the CRC could have important prognostic significance. As reported by Inamura et al., even a minor (50% or less) signet-ring cell component in CRC was associated with higher patient mortality, independent of various tumor molecular and other clinicopathological features. In contrast, mucinous component was not associated with mortality in CRC patients [40]. However, the molecular changes in each subtype of CRC have not been completely understood. For example, TCGA CRC data has 20 mucinous carcinomas, but there is no significant difference of gene expression compared to the adenocarcinomas.

Taken above, using multiple cohorts profile datasets and integrated bioinformatical analysis, we have identified 292 DEGs candidate genes at screening step, and filtered 180 gene nodes in DEGs protein–protein interaction network complex, and finally found 31 mostly changed hub genes, which significant enriched in several pathways, mainly associated with cell cycle process, chemokines and G-protein coupled receptor signaling pathways in colorectal cancer. These findings could significantly improve our understanding of the cause and underlying molecular events in CRC, these candidate genes and pathways could be therapeutic targets for CRC.

## 4. Materials and Methods

### 4.1. Microarray Data Information and DEGs Identification

NCBI-GEO is a free database of microarray/gene profile and next-generation sequencing, from which colorectal cancer and normal or adjacent mucosa tissue gene expression profile of GSE28000, GSE21815, GSE44076 and GSE75970 were obtained. The microarray data of GSE28000 was based on GPL4133 Platforms (Agilent-014850 Whole Human Genome Microarray 4 × 44K G4112F, Agilent Technologies, Santa Clara, CA, USA) and included 43 American Africans (AA) and 43 European Africans (EA) CRC tissues and 40 normal colon tissues (Submission date: 16 March 2011) [7]. The GSE21815 data was based on GPL6480 Platforms (Agilent-014850 Whole Human Genome Microarray 4 × 44K G4112F, Probe Name version, Agilent Technologies) and included 131 CRC tissue and 9 normal colon tissue (Submission date: 13 May 2010) [8,9]. The GSE44076 data was based on GPL13667 Platforms (Affymetrix Human Genome U219 Array, Affymetrix, Santa Clara, CA, USA) and included 98 CRC tissues and 50 healthy colon mucosa (Submission date: 5 Feburary 2013) [10,11]. The GSE75970 data was based on GPL14550 Platforms (Agilent-028004 SurePrint G3 Human GE 8 × 60K Microarray, Agilent Technologies) and included 4 pairs of colorectal cancer tissues and matched paraneoplastic tissues (Submission date: 14 December 2015). We chose these 4 datasets for integrated analysis in this study because these 4 datasets represented five different racial populations: the GSE28000 was generated from the Americans (American Africans and European Africans), the GSE21815 was conducted in Japanese, the GSE44076 data was from Spanish, and the GSE75970 data was conducted in Chinese. This project was approved by Institutional Review Board of Jnining Medical University, and the Code was “JNMC-IRB-20170109”. All procedures of this study were complied with the protocol.

The raw data of high throughput functional genomic expression were integrated for the analysis, the TXT format data were processed in Morpheus Website (Available online: https://software.broadinstitute.org). DEGs were identified with classical *t* test, statistically significant DEGs were defined with *p <* 0.05 and [logFC] > 1 as the cut-off criterion.

For validation, The Cancer Genome Atlas (TCGA) (Available online: https://cancergenome.nih.gov/) colon cancer data was downloaded and analyzed. The TCGA colon cancer data included 22 normal and 122 colon cancers.

### 4.2. Gene Ontology and Pathway Enrichment Analysis

Candidate DEGs functions and pathways enrichment were analyzed using multiple online databases, among them, DAVID is a website with gene annotation, visualization and integrated discovery function, and thus, can provide gene biological meaning. Gene ontology (GO) analysis and pathway analysis were carried out using KEGG PATHWAY (Available online: http://www.genome.jp/kegg), Reactome (Available online: http://www.reactome.org), BioCyc (Available online: http://biocyc.org), Panther (Available online: http://www.pantherdb.org) [12], NHGRI and Gene Ontology website with *p <* 0.05 as the cut-off criterion [13,14,15].

### 4.3. Integration of Protein–Protein Interaction (PPI) Network, Modular Analysis and Significant Candidate Genes and Pathway Identification

First, online database STRING (Available online: http://string-db.org) [16] was employed to develop DEGs-encoded proteins and protein–protein interaction network (PPI). Second, Cytoscape software [17] was utilized to construct protein interaction relationship network and analyze the interaction relationship of the candidate DEGs encoding proteins in colon cancer. Third, the Network Analyzer plug-in was used to calculate node degree, that was, the numbers of inter-connections to filter hub genes of PPI. The corresponding proteins in the central nodes might be the core proteins and key candidate genes that have important physiological regulatory functions.

## 5. Conclusions

Using multiple cohorts profile datasets and integrated bioinformatical analysis, we have identified commonly changed 292 DEGs candidate genes, and finally found 31 mostly changed hub genes, which significant enriched in several pathways, mainly associated with cell cycle process, chemokines and G-protein coupled receptor signaling pathways in colorectal cancer. These findings significantly improve the understanding of the cause and underlying molecular events in colorectal cancer, and the candidate genes and pathways could be used as therapeutic targets.

## Figures and Tables

**Table 1:** 292 differentially expressed genes (DEGs) were identified from four profile datasets, including 165 up-regulated genes and 127 down-regulated genes in the colorectal cancer tissues, compared to normal colon tissues. (The up-regulated genes were listed from the largest to the smallest of fold changes, and down-regulated genes were listed from the smallest to largest of fold changes).

DEGs	Genes Name
Up-regulated	MMP7, *FOXQ1*, *CLDN1*, *KRT23*, *TESC*, *MMP1*, *GDF15*, *ASCL2*, *CXCL3*, *CXCL1*, *CTHRC1*, *TRIB3*, *SLC6A6*, *INHBA*, *CHI3L1*, *MMP11*, *CLDN2*, *SLCO4A1*, *ACSL6*, *NFE2L3*, *FABP6*, *NEBL*, *CXCL2*, *COL10A1*, *AZGP1*, *MACC1*, *CGREF1*, *KLK10*, *GZMB*, *KRT6B*, *TPX2*, *FAP*, *CELSR1*, *PLAU*, *ANLN*, *MMP12*, *CXCL11*, *LRP8*, *ENC1*, *GALNT6*, *SOX9*, *NEK2*, *PMAIP1*, *SLCO1B3*, *GNG4*, *TMEM206*, *EP55*, *CST1*, *SQLE*, *OSBPL3*, *CADPS*, *SERPINB5*, *BIRC5*, *BUB1*, *MMP10*, *TCN1*, *ADAM12*, *S100P*, *NUFIP1*, *SKA3*, *SORD*, *SFRP4*, *SULT2B1*, *CDCA5*, *SIM2*, *ASB9*, *ERCC6L*, *DEFA6*, *MAD2L1*, *RRM2*, *ZNRF3*, *E2F7*, *FAM3B*, *CCNB1*, *EPHB3*, *CDK1*, *SPC25*, *WNT2*, *SLC7A11*, *TNS4*, *NMU*, *CENPF*, *ITGA2*, *PAFAH1B3*, *KIF20A*, *CXCL6*, *MND1*, *CCNA2*, *CCL20*, *FOXA2*, *ODAM*, *DIO2*, *RNF43*, *FERMT1*, *PYCR1*, *RNF183*, *PAQR4*, *GPR143*, *UBE2T*, *BRCA2*, *UNC5CL*, *CKS2*, *RUNX1*, *DBF4*, *CEP72*, *XKRX*, *CDC45*, *KIF18A*, *CENPW*, *FAM150A*, *PPBP*, *RAB15*, *CMTM8*, *FANCI*, *ECE2*, *NOX4*, *ASPM*, *SLC5A1*, *PCSK9*, *TTK*, *FAM64A*, *CKAP2L*, *KDELR3*, *LIPG*, *CDCA2*, *NCAPG*, *KIF15*, *ESCO2*, *PBK*, *NUF2*, *EZH2*, *NUP62CL*, *CDKN3*, *EPHB2*, *KCTD14*, *OXGR1*, *CNPY3*, *GINS2*, *LMNB2*, *DLGAP5*, *PSAT1*, *POLR1D*, *HPDL*, *REG1B*, *CDC25C*, *EXO1*, *ASPHD1*, *LRRC8E*, *SFTA2*, *HMMR*, *GPX2*, *DNAH2*, *HELLS*, *CLCN5*, *SLC12A2*, *RAD51AP1*, *CENPA*, *TRIM29*, *ONECUT2*, *SYNCRIP*, *CENPE*, *PF4*, *CDCA7*, *BACE2*, *CKAP2L*, *KDELR3*, *LIPG*, *CDCA2*, *NCAPG*, *KIF15*, *ESCO2*, *PBK*
Down-regulated	*CLCA4*, *AQP8*, *GUCA2B*, *ZG16*, *CA2*, *CLCA1*, *ITLN1*, *HSD17B2*, *CHP2*, *AKR1B10*, *CEACAM7*, *GCG*, *SLC4A4*, *BEST2*, *LAMA1*, *HRASLS2*, *FCGBP*, *SCARA5*, *HEPACAM2*, *SCNN1B*, *MT1H*, *CDKN2B*, *LDHD*, *SLC26A2*, *UGT2B15*, *HHLA2*, *VSIG2*, *SCIN*, *MUC4*, *GCNT3*, *INSL5*, *SDCBP2*, *MAMDC2*, *BTNL8*, *LRRC19*, *AHCYL2*, *NR3C2*, *CXCL12*, *MT1G*, *PIGR*, *TNFRSF17*, *TMEM100*, *MYOT*, *HSD11B2*, *STMN2*, *SLC17A4*, *NAP1L2*, *TUBAL3*, *MT1E*, *CEACAM1*, *FAM150B*, *BCAS1*, *KIF16B*, *PTPRH*, *GREM2*, *CA12*, *BTNL3*, *FABP1*, *LIFR*, *SYNPO2*, *MB*, *ATP1A2*, *CDH19*, *DENND2A*, *TSPAN7*, *PAPSS2*, *SCUBE2*, *ATP2A3*, *CILP*, *DPT*, *MAOB*, *KIT*, *GFRA2*, *CCL28*, *TCEA3*, *CCL19*, *GHR*, *P2RY1*, *CNTN3*, *PCOLCE2*, *DES*, *PADI2*, *POU2AF1*, *IRF4*, *SIAE*, *MFAP4*, *ANGPTL1*, *CHST5*, *ABI3BP*, *ANO5*, *ABCA8*, *HSPB3*, *SCGN*, *FAM132A*, *CR2*, *STOX2*, *PTGS1*, *PRKCB*, *LPAR1*, *SLC25A23*, *NR3C1*, *NDN*, *SLC17A8*, *PDE2A*, *OLFM1*, *KCNMA1*, *SERTAD4*, *LGI1*, *KIAA2022*, *COL4A6*, *BCHE*, *TOX*, *PRKAA2*, *SETBP1*, *AGTR1*, *MEIS1*, *ASPA*, *ZSCAN18*, *CHRNA3*, *SCN9A*, *SLC9A9*, *SFRP2*, *GNAI1*, *PLP1*, *AKAP12*, *DCLK1*, *PCK1*

**Table 2:** The significant enriched analysis of differentially expressed genes in colorectal cancer.

Term	Description	Count	*p*-Value
**Up-regulated**			
GO:0044699	single-organism process	129	1.10 × 10^−13^
GO:0008283	cell proliferation	41	1.16 × 10^−11^
GO:0005615	extracellular space	32	1.82 × 10^−10^
GO:0044763	single-organism cellular process	114	6.45 × 10^−10^
GO:0065007	biological regulation	110	3.35 × 10^−9^
GO:0005576	extracellular region	60	5.56 × 10^−9^
GO:0000278	mitotic cell cycle	24	1.51 × 10^−8^
GO:1903047	mitotic cell cycle process	23	1.68 × 10^−8^
GO:0044421	extracellular region part	53	1.74 × 10^−8^
GO:0009987	cellular process	129	2.59 × 10^−8^
**Down-regulated**			
GO:0044699	single-organism process	106	6.94 × 10^−16^
GO:0005488	binding	110	1.68 × 10^−15^
GO:0044464	cell part	115	2.24 × 10^−14^
GO:0005623	cell	115	2.65 × 10^−14^
GO:0043226	organelle	103	6.36 × 10^−14^
GO:0051179	localization	66	7.96 × 10^−14^
GO:0005515	protein binding	92	8.65 × 10^−13^
GO:0044763	single-organism cellular process	96	1.03 × 10^−12^
GO:0043227	membrane-bounded organelle	97	1.58 × 10^−12^
GO:1903047	mitotic cell cycle process	23	6.85 × 10^−11^

**Table 3:** Signaling pathway enrichment analysis of differentially expressed genes function in colorectal cancer.

Pathway	Name	Gene Count	*p*-Value	Genes
**Up-regulated DEG**				
Reactome:R-HSA-418594	G α (i) signalling events	11	8.21 × 10^−7^	*CXCL2 NMU CXCL6 CCL28 CXCL3 PPBP OXGR1 GNG4 CXCL11 INSL5 LPAR1*
Reactome:R-HSA-500792	GPCR ligand binding	14	2.10 × 10^−6^	*CXCL2 WNT2 NMU GCG CXCL6 CCL28 CXCL3 PPBP OXGR1 GNG4 CXCL11 P2RY1 INSL5 LPAR1*
Reactome:R-HSA-380108	Chemokine receptors bind chemokines	6	3.62 × 10^−6^	*CXCL2 CXCL11 CXCL3 CXCL6 CCL28 PPBP*
Reactome:R-HSA-373076	Class A/1 (Rhodopsin-ike receptors)	11	1.24 × 10^−5^	*CXCL2 NMU CXCL6 CCL28 CXCL3 PPBP OXGR1 CXCL11 P2RY1 INSL5 LPAR1*
Reactome:R-HSA-1640170	Cell Cycle	15	1.36 × 10^−5^	*DBF4 TPX2 BIRC5 CEP72 CENPA CENPF MND1 GINS2 CENPE BRCA2 CDC25C MAD2L1 BUB1 RRM2 TUBAL3*
Reactome:R-HSA-2500257	Resolution of Sister Chromatid Cohesion	7	1.64 × 10^−5^	*BIRC5 CENPA CENPF CENPE MAD2L1 BUB1 TUBAL3*
Reactome:R-HSA-68877	Mitotic Prometaphase	7	2.45 × 10^−5^	*BIRC5 CENPA CENPF CENPE MAD2L1 BUB1 TUBAL3*
Reactome:R-HSA-5663220	RHO GTPases Activate Formins	7	3.12 × 10^−5^	*BIRC5 CENPA CENPF CENPE MAD2L1 BUB1 TUBAL3*
Reactome:R-HSA-69278	Cell Cycle, Mitotic	13	3.23 × 10^−5^	*DBF4 TPX2 BIRC5 CEP72 CENPA CENPF GINS2 CENPE CDC25C MAD2L1 BUB1 RRM2 TUBAL3*
Reactome:R-HSA-375276	Peptide ligand-binding receptors	8	5.24 × 10^−5^	*CXCL2 NMUCXCL6 CCL28 CXCL3 PPBP CXCL11 INSL5*
KEGG Pathway:hsa04060	Cytokine-cytokine receptor interaction	9	7.61 × 10^−5^	*TNFRSF17 CXCL2 LIFR INHBA CXCL6 CCL28 CXCL3 PPBP CXCL11*
**Down-regulated DEGs**				
Reactome:R-HSA-1640170	Cell Cycle	18	1.83 × 10^−9^	*CDC45 NCAPG KIF20A NEK2 KIF18A CCNB1 NUF2 CCNA2 CDCA5 CDKN2B SPC25 PRKCB CDK1 ESCO2 EXO1 ERCC6L CENPW*
Reactome:R-HSA-69278	Cell Cycle, Mitotic	16	5.78 × 10^−9^	*HMMR CDC45 NCAPG KIF20A NEK2 KIF18A CCNB1 NUF2 CCNA2 CDCA5 CDKN2B SPC25 PRKCB CDK1 ESCO2 ERCC6L*
Reactome:R-HSA-68877	Mitotic Prometaphase	8	3.19 × 10^−7^	*NCAPG KIF18A CCNB1 NUF2 CDCA5 SPC25 CDK1 ERCC6L*
Reactome:R-HSA-2500257	Resolution of Sister Chromatid Cohesion	7	2.74 × 10^−6^	*KIF18A CCNB1 NUF2 CDCA5 SPC25 CDK1 ERCC6L*
KEGG PathwaY:hsa04960	Aldosterone-regulated sodium reabsorption	5	3.09 × 10^−6^	*ATP1A2 NR3C2 PRKCB SCNN1B HSD11B2*
Reactome:R-HSA-68886	M Phase	10	4.77 × 10^−6^	*NCAPG KIF20A KIF18A CCNB1 NUF2 CDCA5 SPC25 PRKCB CDK1 ERCC6L*
Reactome:R-HSA-380108	Chemokine receptors bind chemokines	5	1.48 × 10^−5^	*CXCL12 CCL20 PF4 CXCL1 CCL19*
Reactome:R-HSA-2514853	Condensation of Prometaphase Chromosomes	3	4.06 × 10^−5^	*NCAPG CCNB1 CDK1*
KEGG Pathway:hsa04110	Cell cycle	6	4.53 × 10^−5^	*CDC45 TTK CCNB1 CCNA2 CDKN2B CDK1*
KEGG Pathway:hsa04062	Chemokine signaling pathway	7	5.37 × 10^−5^	*CXCL12 CCL20 GNAI1 PF4 CXCL1 PRKCB CCL19*
Reactome:R-HSA-4419969	Depolymerisation of the Nuclear Lamina	3	6.20 × 10^−5^	*CCNB1 PRKCB CDK1*

**Table 4:** Pathway enrichment analysis of Module 1 genes function.

Term	Description	Count	*p*-Value
R-HSA-68877	Mitotic Prometaphase	12	2.56 × 10^−24^
GO:0000819	sister chromatid segregation	13	5.52 × 10^−24^
GO:0000280	nuclear division	15	2.13 × 10^−23^
GO:0048285	organelle fission	15	5.03 × 10^−23^
GO:1903047	mitotic cell cycle process	16	8.44 × 10^−23^
GO:0098813	nuclear chromosome segregation	13	8.98 × 10^−23^
GO:0007067	mitotic nuclear division	14	1.40 × 10^−22^
GO:0022402	cell cycle process	17	1.70 × 10^−22^
GO:0000278	mitotic cell cycle	16	2.87 × 10^−22^
R-HSA-2500257	Resolution of Sister Chromatid Cohesion	11	4.68 × 10^−22^
GO:0007059	chromosome segregation	13	6.23 × 10^−22^
R-HSA-69278	Cell Cycle, Mitotic	14	1.32 × 10^−21^
GO:0007049	cell cycle	17	1.11 × 10^−20^
R-HSA-1640170	Cell Cycle	14	1.84 × 10^−20^
GO:0000775	chromosome, centromeric region	11	4.37 × 10^−20^
R-HSA-68886	M Phase	12	1.09 × 10^−19^
GO:0000793	condensed chromosome	11	1.41 × 10^−19^
GO:0098687	chromosomal region	12	2.49 × 10^−19^
GO:0000776	kinetochore	10	2.70 × 10^−19^
GO:0000070	mitotic sister chromatid segregation	10	3.14 × 10^−19^
GO:0051301	cell division	13	6.90 × 10^−19^
GO:0000777	condensed chromosome kinetochore	9	7.74 × 10^−18^
GO:0000779	condensed chromosome, centromeric region	9	1.64 × 10^−17^
GO:0044427	chromosomal part	13	6.36 × 10^−17^
GO:0007062	sister chromatid cohesion	9	8.20 × 10^−17^
GO:0051276	chromosome organization	14	1.02 × 10^−16^
GO:0051983	regulation of chromosome segregation	8	2.39 × 10^−16^
GO:0005694	chromosome	13	3.03 × 10^−16^
GO:1902589	single-organism organelle organization	15	4.31 × 10^−16^
GO:0007346	regulation of mitotic cell cycle	11	1.03 × 10^−15^

**Table 5:** Pathway enrichment analysis of Module 2 genes function.

Term	Description	Count	*p*-Value
R-HSA-418594	G α (i) signaling events	14	1.36 × 10^−27^
GO:0008009	chemokine activity	10	6.81 × 10^−24^
GO:0042379	chemokine receptor binding	10	3.15 × 10^−23^
R-HSA-380108	Chemokine receptors bind chemokines	10	3.15 × 10^−23^
R-HSA-373076	Class A/1 (Rhodopsin-like receptors)	13	3.87 × 10^−23^
GO:0001664	G-protein coupled receptor binding	12	8.63 × 10^−22^
R-HSA-500792	GPCR ligand binding	13	2.51 × 10^−21^
hsa04062	Chemokine signaling pathway	11	8.22 × 10^−21^
R-HSA-375276	Peptide ligand-binding receptors	11	1.08 × 10^−20^
GO:0060326	cell chemotaxis	11	6.67 × 10^−20^
GO:0002685	regulation of leukocyte migration	10	2.42 × 10^−19^
R-HSA-388396	GPCR downstream signaling	14	2.58 × 10^−19^
GO:0070098	chemokine-mediated signaling pathway	9	2.86 × 10^−19^
GO:0045236	CXCR chemokine receptor binding	7	1.26 × 10^−18^
GO:0002687	positive regulation of leukocyte migration	9	4.32 × 10^−18^
GO:0007186	G-protein coupled receptor signaling pathway	14	7.31 × 10^−18^
GO:0050921	positive regulation of chemotaxis	9	9.82 × 10^−18^
GO:0005125	cytokine activity	10	1.07 × 10^−17^
R-HSA-372790	Signaling by GPCR	14	1.20 × 10^−17^
hsa04060	Cytokine-cytokine receptor interaction	10	6.02 × 10^−17^
GO:0005126	cytokine receptor binding	10	8.95 × 10^−17^
GO:0002690	positive regulation of leukocyte chemotaxis	8	1.25 × 10^−16^
GO:0030595	leukocyte chemotaxis	9	2.93 × 10^−16^
GO:0050920	regulation of chemotaxis	9	3.23 × 10^−16^
GO:0002688	regulation of leukocyte chemotaxis	8	4.83 × 10^−16^
GO:0006935	chemotaxis	11	5.74 × 10^−16^
GO:0042330	taxis	11	5.86 × 10^−16^
GO:0050900	leukocyte migration	10	1.08 × 10^−15^
GO:0030335	positive regulation of cell migration	10	2.69 × 10^−15^
GO:2000147	positive regulation of cell motility	10	3.62 × 10^−15^

## Supplementary Materials

Supplementary materials can be found at www.mdpi.com/1422-0067/18/4/722/s1.

## Abbreviations

CRC	Colorectal cancer
DEG	Differentially expressed genes
PPI	Protein–protein interaction network
GPCR	G-protein couple receptor
