# Supplementary material for: Identification of Key Candidate Genes and Pathways in Colorectal Cancer by Integrated Bioinformatical Analysis
Source: Int J Mol Sci. 2017 Mar 28;18(4):722. doi: 10.3390/ijms18040722 (PMC5412308; doi:10.3390/ijms18040722)
Supplement: Supplementary file 1 [file ijms-18-00722-s001.pdf]

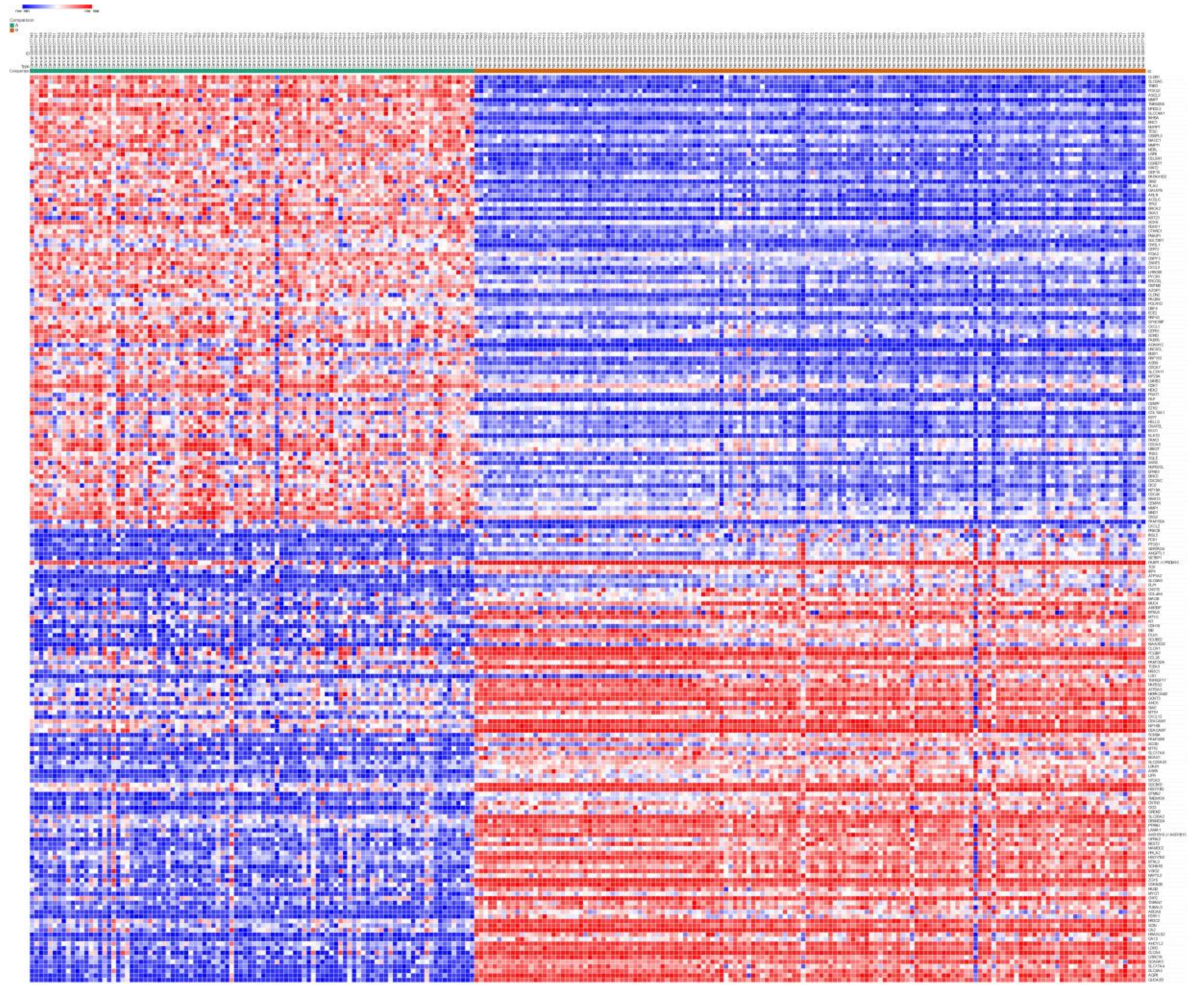

Figure S1. Expression heat map of the commonly changed 292 DEGs. Red color meant up-regulated genes, and the blue color meant down-regulated genes.

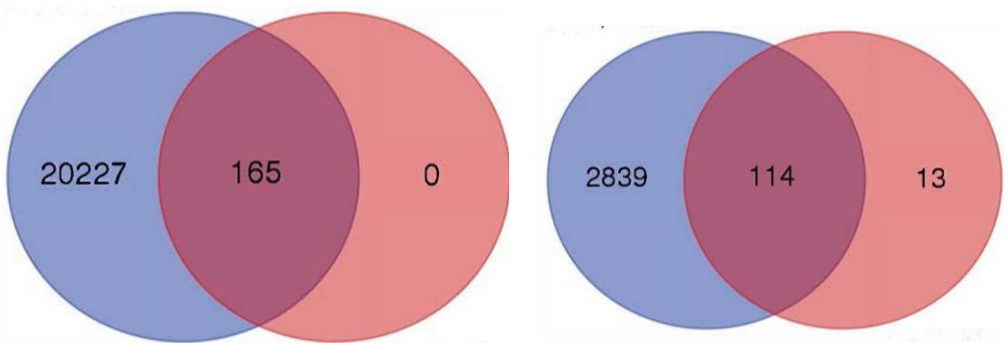

(**a**) (**b**)

Figure S2. Commonly up-regulated genes (165 DEGs, **A**) and down-regulated genes (114 DEGs, **B**) in TCGA colon adenocarcinoma data and the four datasets from this study.

Table S1. The commonly up-regulated and down-regulated gene in the four datasets from this study and in the TCGA dataset of colon cancers.

Names	Total	Elements
Up-expression in Colon Adenocarcinoma vs. Normal Up-regulated DEGs	165	MMP7 SFTA2 UNC5CL SQLE RNF43 ZNRF3 CDC45 CDCA2 KCTD14 EZH2 SLC12A2 CDCA7 DNAH2 SKA3 RRM2 POLR1D CXCL1 DEFA6 CDKN3 GDF15 BIRC5 NEBL PAFAH1B3 FAM3B NUF2 LIPG FABP6 CLDN1 ASB9 EPHB2 S100P NUP62CL CDC25C CADPS NOX4 ASPHD1 SLCO4A1 FAP SPC25 CXCL6 KIF20A COL10A1 TTK NCAPG TRIB3 LMNB2 XKRX GPX2 E2F7 MMP1 CXCL2 HPDL FOXQ1 RUNX1 DBF4 REG1B KDELR3 BUB1 MND1 OXGR1 HELLS TNS4 KLK10 ERCC6L TESC KRT23 ADAM12 ANLN PF4 CHI3L1 NUFIP1 UBE2T CENPE WNT2 CEP55 SYNCRIP PMAIP1 NMU MMP10 GINS2 CXCL11 ODAM CCL20 KIF15 FERMT1 SLC5A1 CXCL3 CDK1 PPBP MAD2L1 ITGA2 CCNA2 CKS2 GALNT6 FAM150A LRP8 CENPW NFE2L3 SIM2 EXO1 NEK2 TMEM206 ECE2 PYCR1 CENPA TCN1 PSAT1 ACSL6 KRT6B FAM64A SLC6A6 BACE2 OSBPL3 EPHB3 SLC7A11 MMP12 CDCA5 MMP11 MACC1 ESCO2 SLCO1B3 SFRP4 PBK ASPM INHBA CLCN5 SORD RNF183 CCNB1 PAQR4 SOX9 ASCL2 RAB15 CEP72 PCSK9 BRCA2 CNPY3 DIO2 CKAP2L CLDN2 CST1 AZGP1 FANCI KIF18A ENC1 CTHRC1 CENPF CMTM8 TPX2 GPR143 FOXA2 TRIM29 LRRC8E CGREF1 GZMB DLGAP5 RAD51AP1 SULT2B1 PLAU SERPINB5 GNG4 CELSR1 HMMR ONECUT2
Under-expression in Colon Adenocarcinoma vs. Normal Down-regulated DEGs	114	KCNMA1 HSD17B2 NAP1L2 HSD11B2 PRKCB PAPSS2 ANO5 GHR CCL19 BCHE SLC9A9 TSPAN7 HHLA2 AHCYL2 FCGBP PTPRH MT1G SFRP2 AQP8 KIT TNFRSF17 BTNL3 SLC17A4 TOX LRRC19 SCNN1B DENND2A CDKN2B LAMA1 SETBP1 GFRA2 CA12 ATP2A3 ANGPTL1 GREM2 NR3C2 MT1E SERTAD4 GNAI1 FAM132A AKAP12 HRASLS2 SLC26A2 COL4A6 CHRNA3 GCNT3 CDH19 VSIG2 SIAE CEACAM1 PLP1 STMN2 PCOLCE2 SLC25A23 SLC4A4 ZSCAN18 CILP LDHD SYNPO2 KIAA2022 TUBAL3 FAM150B POU2AF1 HSPB3 SCIN DCLK1 KIF16B MYOT OLFM1 STOX2 NR3C1 ABCA8 P2RY1 INSL5 DES ASPA SCGN PCK1 ABI3BP MUC4 TMEM100 IRF4 CNTN3 PRKAA2 DPT MFAP4 GUCA2B PDE2A CHP2 SCARA5 MAOB CLCA4 ATP1A2 MB LIFR CXCL12 LGI1 CA2 BCAS1 PADI2 BEST2 GCG SCUBE2 CHST5 TCEA3 MT1H HEPACAM2 PTGS1 LPAR1 MAMDC2 SDCBP2 SLC17A8 SCN9A NDN
